# Development of Intrinsic Seismic Vulnerability Index (ISVI) for assessing roadway system and its assets framework

**DOI:** 10.1016/j.mex.2022.101818

**Published:** 2022-08-12

**Authors:** Ahmad Mohamad El‐Maissi, Sotirios A. Argyroudis, Moustafa Moufid Kassem, Fadzli Mohamed Nazri

**Affiliations:** a School of Civil Engineering, Engineering Campus, Universiti Sains Malaysia, Penang 14300, Malaysia; b Department of Civil and Environmental Engineering, College of Engineering, Design and Physical Sciences, Brunel University, London UB8 3PH, United Kingdom; c Academic Fellow, School of Civil Engineering, Engineering Campus, Universiti Sains Malaysia, Penang 14300, Malaysia

**Keywords:** Intrinsic seismic vulnerability index, Accessibility Index, Disaster risk reduction, Resilient infrastructure, Vulnerability assessment, Critical service centers

## Abstract

Roadway systems and their assets are the backbone of the transport sector and are vital for social and economic prosperity. Hence, it is important to design and develop transportation networks that can withstand natural hazards such as earthquakes. In recent decades, research concerning disaster risk management for roadway systems has received a lot of attention, particularly via the use of seismic vulnerability assessment methods. The majority of those models focus on a single criterion e.g., physical degradation of road assets, traffic disturbance, and/or functionality loss of the network, rather than considering how different criteria interact, such as association between asset damage, functionality, and network traffic. The main purpose of this study is to provide an integrated methodology for evaluating the seismic vulnerability of road networks to inform decision-making for risk mitigation. The proposed framework correlates the Intrinsic Seismic Vulnerability Index (ISVI) scores with the variation of accessibility rates to critical service centers. The methodology is demonstrated through an application to a part of a road network for specific seismic scenarios. The ISVI quantifies the impact caused by a parameter's physical performance on the road behaviour using Non-Linear Dynamic Analysis (NLDA) technique, which can reduce or limit the role of the studies that are based on expert opinion decisions. The validated results shows that the embankment height is considered the most effective parameter in the physical assessment approach, followed by the number of lanes, while the soil type and pavement strength are the least effective parameters with a better effectiveness for soil type compared to pavement strength. Additionally, the integration between the physical assessment approach and the analysed accessibility rates is clearly showing compatibility between the vulnerability and accessibility approach, demonstrating more precise assessment tool by considering the correlation between the vulnerability rates and the reduced accessibility levels. The proposed approach can assist infrastructure owners and operators to reduce risk and boost emergency accessibility.

• Conducting ISVI for roadway and its assets based on physical damage approach.

• Assessing road networks accessibility rates by introducing an accessibility index (AI) using different geographical aspects.

• Formulating an integrated model between physical damage and traffic accessibility through building transport performance maps.

## Graphical abstract

**Figure:**
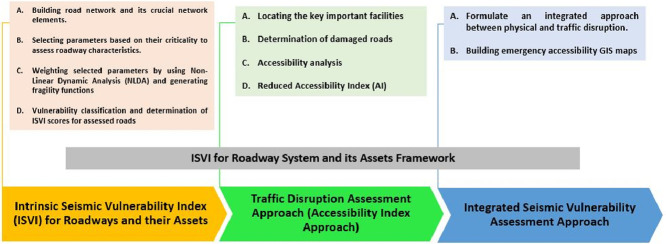

**Specifications table**

**Table:** 

Subject area:	Engineering
More specific subject area:	*Integrating Traffic and Earthquake Models*
Name of your method:	*Intrinsic Seismic Vulnerability Index (ISVI) for Assessing Roadway System and its Assets.*
Name and reference of original method:	S. Adafer and M. Bensaibi, "Seismic vulnerability classification of roads," *Energy Procedia,* vol. 139, pp. 624-630, 2017.
Resource availability:	*Not Applicable*

## Background

Recent earthquakes, that had a significant impact on human life and economic development, have raised worries about the robustness of road networks and their components exposed to seismic hazards. As a result, various approaches for evaluating the vulnerability and mitigating seismic risk for these systems have been developed. Vulnerability assessments at the asset level consider the degree of damage to a specific asset (e.g. a bridge) by using fragility functions and/or vulnerability indexes, whereas studies at the network level take into consideration the functionality of the network by studying traffic-related aspects such as the accessibility or link importance [1]. Previous studies assessed the vulnerability of road networks and their components using multi-criteria characteristics of roadway system and its assets, without taking into account the relation between physical damage, traffic disruption, and road network functionality [2]. Subsequently, different methodologies that examine the reliability and serviceability of roadway systems subjected to earthquakes have been developed, but most of them focus solely on the likelihood of physical or functional failure of the assets and provide no information on the role of these assets in the roadway system [3,4].

**Fig. 1:**
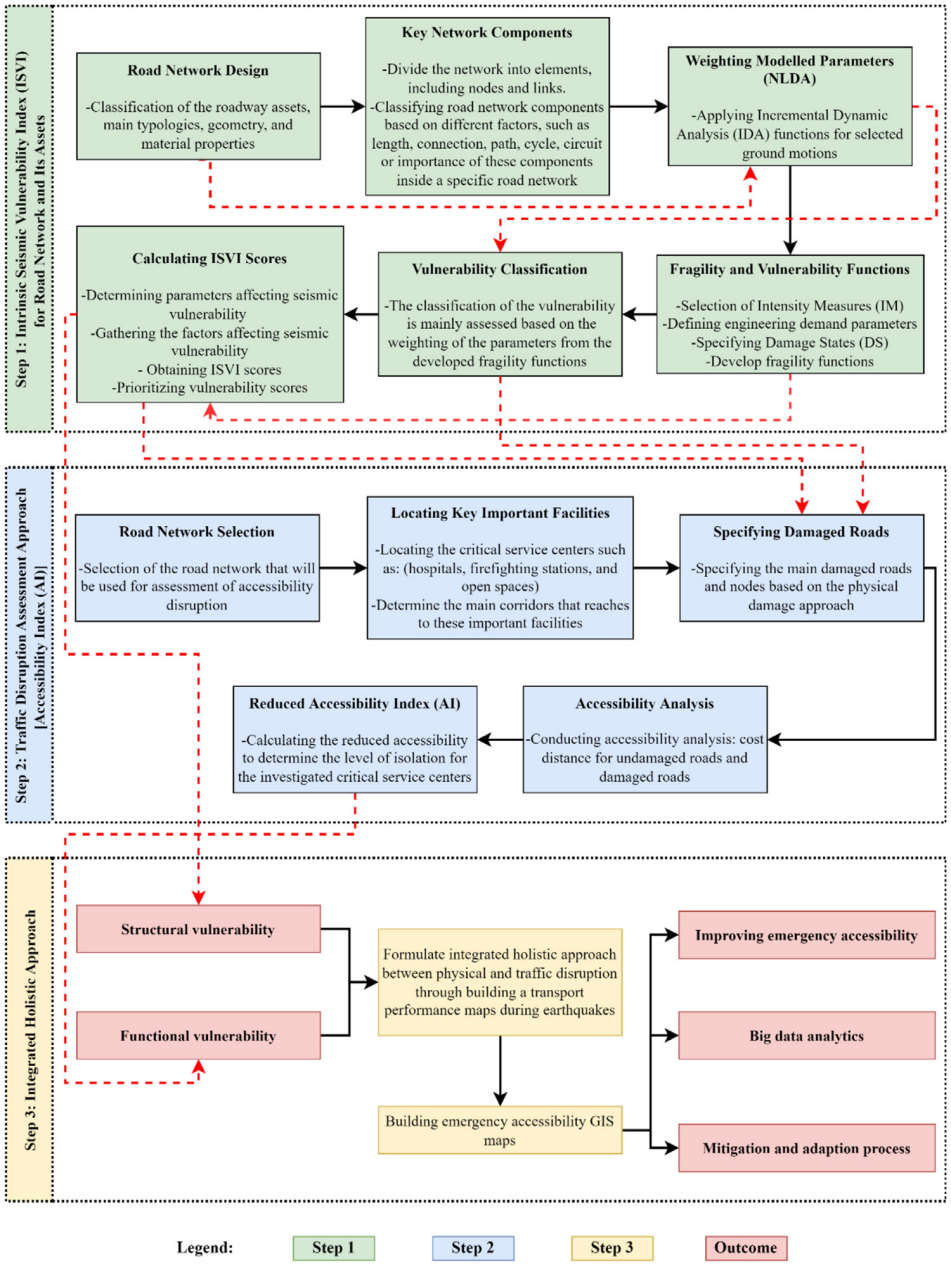
Intrinsic Seismic Vulnerability Index (ISVI) for Assessing Roadway Systems.

The present paper introduces a methodology that includes a modified Intrinsic Seismic Vulnerability Index (ISVI) for the road network and its components, which is then integrated with the road network functionality to evaluate accessibility rates. This framework focus on the earthquake vulnerability assessment of roadway and its assets based on the minimum identified scale of a specific city. Identifying this scale is considered a crucial step because the assessment procedure is considered complex and needs to be precise, where the challenge lies in identifying the minimum possible scale of expression of the city and its elements to assess the vulnerability of roadway system. This scale can be categorized into two main levels, the macro-level, which considers the whole city development plan, and the micro-level, which focuses on the road network itself. The developed framework is built on the basis of the micro-level, where three main steps are introduced in the proposed method as illustrated in Fig.1 and described in the following. In some cases, illustrative examples from the case study Penang area, Malaysia are provided. This case study is considered the baseline for validating this methodology and is described in the method validation section.

In **Step 1**, the roadway network is constructed with respect to the main classified assets, by taking into account different factors, for instance: node-to-node connectivity, roadway length, and the role of the assets in the network. The Intrinsic Seismic Vulnerability Index (ISVI) is then evaluated based on critical vulnerability parameters, namely the embankment height, pavement strength, ground condition, and road width. The vulnerability is then categorized into three main classes based on the range of the estimated ISVI scores. In particular, the road segment is classified as safe, moderately resistant, and low resistant. This is followed by weighting the investigated parameter based on the developed Non-Linear Dynamic Analysis (NLDA) model. The latter is employed to develop the fragility functions that are utilized to evaluate these simulated parameters. This step helps in prioritizing the investigated parameters, where the extracted results from the NLDA are used to weight the parameters by using Analytical Hierarchy Process (AHP). Subsequently, the ISVI scores are calculated from these weighted parameters based on their effectiveness, where the most effective parameter is defined based on the difference of damage probability between the two transmission stages at collapse state as described in Step 1.
In **Step 2**, the obtained ISVI values for the main assessed roadways are used to develop the traffic disruption approach, by determining the Accessibility Index (AI) of road networks. This step is targeting to the identification of the main corridors that can be used in case of emergency for accessing critical service centers after earthquake incidents.
Finally, **Step 3**, forms an integrated approach between physical (Step 1) and traffic-based (Step 2) approaches, where the transportation performance for the area under investigation is mapped, by combining the ISVI and AI outcomes. This step highlights the reduced network accessibility and the accessibility of the critical service centers and provides an integrated tool for obtaining big data analytics that facilitate efficient natural disaster security measures and solutions, and hence reduction of losses.

In Fig.1 the black arrows represent the direct relation between the implemented steps in the same category of the methodology, while the red dotted arrows are used to represent the indirect correlation between the implemented steps in the same category or different category.

**Step 1: Intrinsic Seismic Vulnerability Index (ISVI) for roadways and their assets**

The Intrinsic Seismic Vulnerability Index (ISVI) is established through building an analytical evaluation approach that considers four important parameters for roads and their investigated parameters (embankments height, ground condition, pavement strength, and road width). The parameters are evaluated based on their relevant criticality as indicated in prior research [3]. The method involves weighting roadway parameters using an analytical approach that takes into consideration the variation of improvement percentage between the investigated parameters. The latter is calculated based on the concept of resistance design for roadway systems, where the variation of improvement is extracted by calculating the probability of damage difference between the two transmission stages at the collapse states at collapse state by employing fragility functions.

Probabilistic damage distribution *functions* are commonly used to assess the physical vulnerability of roadway assets for a given Intensity Measure (IM). A cumulative damage distribution function is represented by fragility curves, which correlate the seismic IM and the probability of exceeding different damage states of the infrastructure asset [1]. Fragility functions are commonly developed based on numerical approaches, by which the performance of the damaged road under the effect of seismic loads is quantified, using practical and efficient Engineering Demand Parameters (EDP), such as the maximum vertical displacement or settlement at the road or embankment surface [1]. A fragility function also represents a seismic risk assessment indicator, which is considered as an efficient tool in decision making for retrofitting, identifying the damage cost, and preventing loss of life during seismic events.

**Fig. 2:**
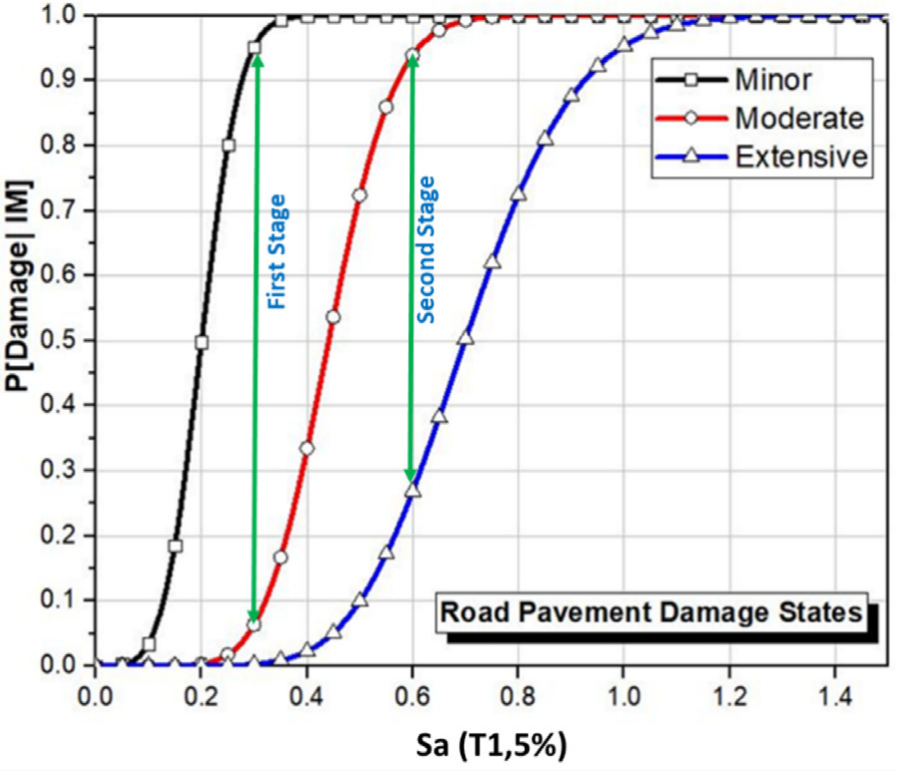
Example of Fragility Functions for Road Pavements and its Main Transmission Stages Reproduced by El Maissi et al. [5].

The degradation of the main parameters of the roadway can be quantified by defining the probability of damage at collapse stage for a specific seismic intensity. To improve the overall reliability of roadways and their assets, the resistance design concept is primarily evaluated for two main transmission stages. As demonstrated in Fig. 2, the first stage is investigated based on the variation between minor and moderate damage states, on the other hand, the second stage is assessed on the basis of the variation between moderate and extensive damage states. Previous research is used as the baseline to determine the main vulnerability categories of the investigated parameters [3,4]. This analytical approach emphasises the severe damage, which could affect the assessed roadway system and its assets and helps in calculating and prioritizing the vulnerability parameters from the most influential to the least.

After ranking the investigated parameters with respect to their effectiveness, the Analytical Hierarchy Process (AHP) is used to give a specific weight $\;(W_{i})$ for each parameter. Additionally, the scores $\;(S_{i})\;$ for the main evaluated parameters are determined and categorised using the assessment criteria by Adafer and Bensaibi [3]. Equation 1 is used to determine the Intrinsic Seismic Vulnerability Index (ISVI) for the roadway system.

(1) $$\text{ISVI}=\sum_{i=1}^{n}S_{i}\times WP_{a_{i}}$$

Where $S_{i}\;$ symbolizes the scoring values of the main assessed parameters; $WP_{a_{i}}\;$ is the i-th of four weighted parameters with respect to the variation of the percentage of improvement outcomes; and n presents the total number of parameters.

**Fig. 3:**
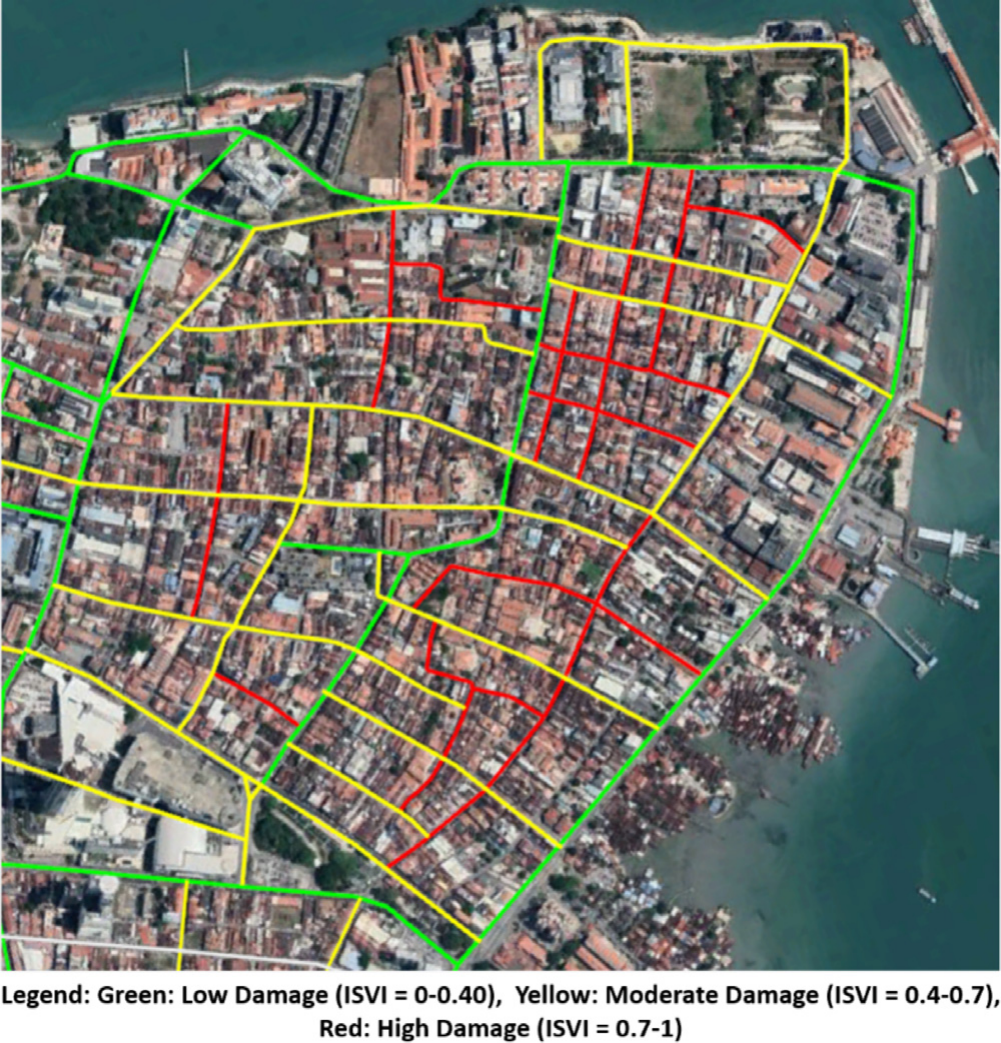
Classified Damaged Roadways for earthquake incidents with high seismic intensity (IX) based on ISVI Scores.

Subsequently, the calculated ISVI scores are used to categorize the investigated roadways under earthquake effect at seismic intensity of IX, where these roadways are considered as low damage (ISVI = 0-0.4), moderate damage (ISVI = 0.4-0.7), or high damage (ISVI = 0.7-1). The map is created at a critical seismic intensity (IX), because the ISVI values at the two different seismic intensities show minor differences. Moreover, the IX intensity provides greater critical damage ranges (the most critical and disastrous situation that should be focused on). The map in Fig. 3 illustrates the categorised damaged roadways during earthquakes.

## Mapping of the road network and its crucial elements

**Fig. 4:**
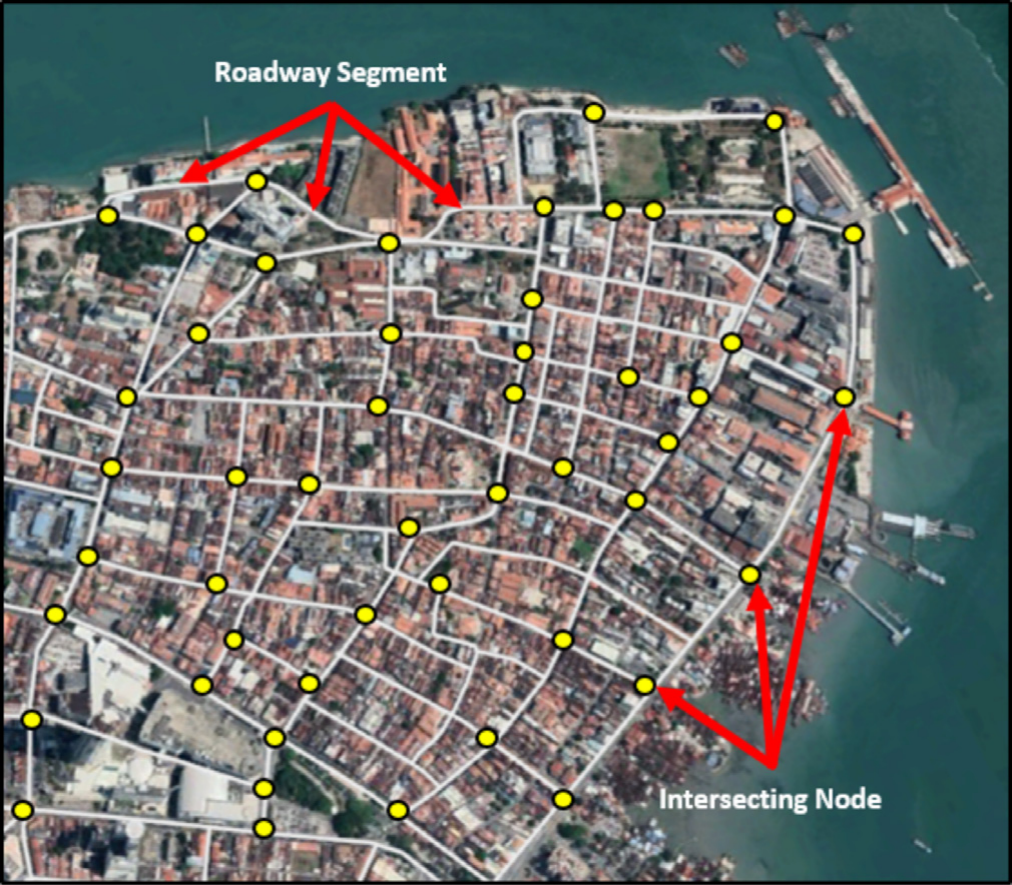
Specified Crucial Elements for the Investigated Road Network.

Road networks are specified based on various factors, such as the roadway length, connections between roadways, circuit, manoeuvring path for vehicles, and the criticality of these elements in a specific network. For instance, Kilanitis and Sextos [2] divided the network into links (roadway elements), nodes (intersecting points between different roadways), and crucial network elements (tunnels and bridges). Nevertheless, this framework takes into consideration the functionality of the road network that is defined in terms of accessibility. To put it another way, a roadway is considered accessible if it is linked (structurally) and accessible (functionally). The actual physical degradation of the roadway and its assets is a significant factor in deciding traffic accessibility and functionality. The vulnerability evaluation does not look at the entire road network; instead, it focuses on the most susceptible weak locations. Hence, it is necessary to break every network into small pieces, including roadways and the intersecting points between them, in order to assess the seismic risk. Nodes represent the point elements, where the user can enter from (e.g., intersections and bridges) or change the direction of travel (e.g., ramps, road interchanges, and roundabouts). The linear pieces that connect these nodes are referred to as links. All the specified crucial elements that are used to build the road network are illustrated in Fig.4.

## Selecting parameters based on their criticality to assess roadway characteristics

**Fig. 5:**
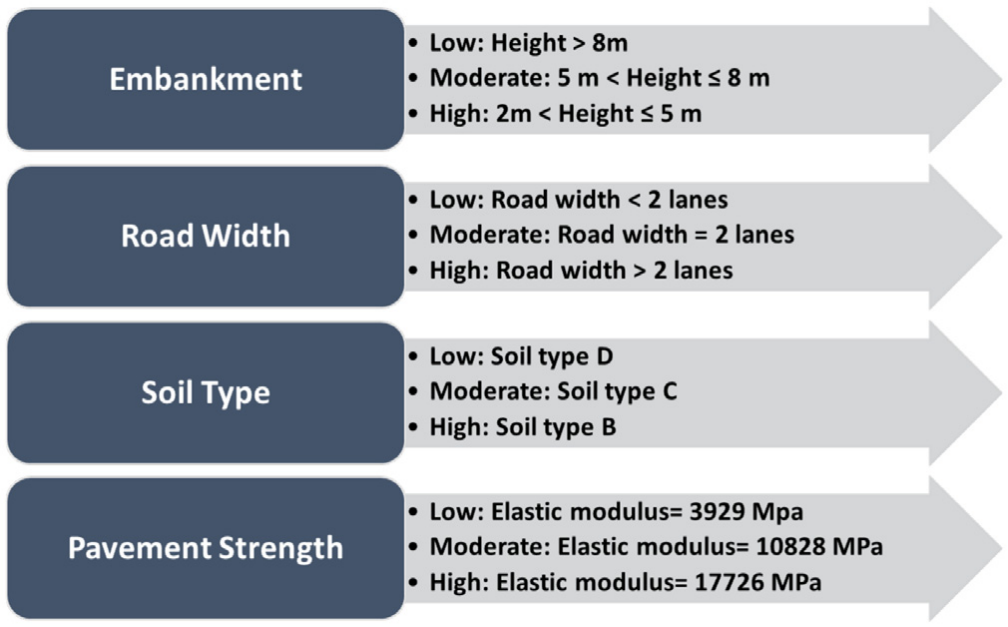
Classified Parameters with respect to Different Vulnerability Classes [3,4]

The seismic and geotechnical qualities of the studied region, as well as other physical and geometric aspects, influence the roadway vulnerability. The main investigated parameters used in this framework were selected based on previous research that described and prioritised their criticality, and how these parameters affect the roadway system during earthquakes. For instance, Maruyama et al. [6] concluded that the embankments are considered one of the roadway system's most influenced assets (critical asset) during earthquakes, by which, more than 65% of the destroyed assets are related to the damaged embankments accounts. Moreover, Adafer and Bensaibi [3] investigated the Intrinsic Seismic Vulnerability Index for the roadways in Algeria, where the results of this research has shown that embankment height is considered the most effective parameter followed by the soil type, road width, and pavement strength. Nevertheless, many other studies investigated different parameters (maintenance conditions, roadway redundancy, and liquefaction potential). Due to these facts these four parameters are selected to develop the seismic assessment models in this framework. This is followed by categorizing the selected parameters into three different vulnerability classes (Low, Moderate, and High) as shown in Fig. 5.

## Weighting selected parameters by using Non-Linear Dynamic Analysis (NLDA) and generating fragility functions

As a result of the insufficient data and expert opinion used to build the seismic vulnerability index, empirical methodologies associated by uncertainty. On the contrary, analytical methods can give more accurate and reliable vulnerability evaluations [7]. Non-Linear Dynamic Analysis (NLDA) is employed in this framework to analyse the structural characteristics of a specific assets and its effect on roadway system and to extract the probability of damage that is used in weighting the investigated parameters (i.e., road width, pavement strength, soil type), which determine the Intrinsic Seismic Vulnerability Index (ISVI) scores.

Different scenarios are considered in developing the assessment model for each asset. For instance, when assessing the probability of damage based on the embankment height, different height values h=4 m, or h=6m or h=9m are considered, while the other parameters (road width, pavement strength, and soil type) are fixed at a specific value. The worst-case scenarios that have the most critical impact are finally included in the model. The same process is repeated to assess all the main parameters on the roadway system.

**Fig. 6:**
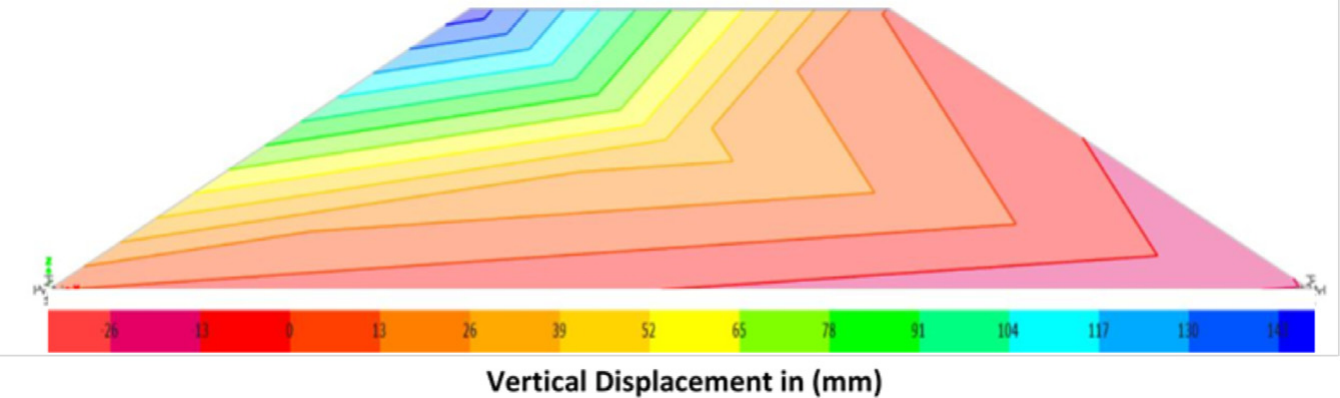
Vertical Displacement Movement of an Embankment Model under Seismic and Traffic Load Combination.

To sum up, traffic and earthquakes loadings are two kinds of loads with different natures and should be combined in seismic assessment of single parameter. The destructive power and duration of earthquakes are greater than that of vehicles on roadways. Therefore, it is proper to consider earthquake as the condition for load combination. Since the methodology in this paper is based on the condition of earthquake, the whole load combination is divided into those with time history analysis and traffic loadings. Fig. 6 shows a sample of embankment analysis in case of load combination of seismic and traffic loading.

The developed analytical approach that is used in this methodology is built by using the four determined parameters solely by referring to their classification in the vulnerability classes that is described in Section 2.2. All of this is employed through the Incremental Dynamic Analysis (IDA) [8]. The vertical settlement is taken as the main Engineering Demand Parameters (EDP) to classify the Damage States (DS) of the roadway system. The DS are classified into four main categories based on the vertical settlement results extracted from the conducted model as follows: None (No settlement), minor (Slight settlement less than 30cm), moderate (Moderate settlement between 30 and 50 cm), and extensive (High settlement larger than 60cm). Additionally, seven ground motions are precisely chosen and extracted from different online databases Consortium of Organizations for Strong-Motion Observation Systems Database (COSMOS), and Pacific Earthquake Engineering Research (PEER) to obtain the Non-Linear Time History Analysis (NL-THA).

This is followed by defining the Intensity Measure (IM) by using the spectral acceleration as a fundamental period Sa (T1, 5%). The spectral acceleration (Sa) for the vibration period (T1) is related to the critical deformed shape in the road structural element of the 1st mode shape with a 5% damping ratio and is considered the most appropriate measure in relevant research [9], [10], [11]. The first mode spectral acceleration is considered a reliable indicator of a structure's ability to respond elastically, it is frequently utilized as the baseline earthquake intensity scale factor in time-history research. The employment of the cumulative distribution function (CDF) of a specific infrastructure performance damage state, can help in the formulation of seismic vulnerability assessments of road networks with respect to DM and IM [1].

Eq. 2, is used to conduct the fragility functions, as formulated by Ibrahim and El-Shami [12] and Kassem et al. [13].

(2) $$P\left[\text{Damage}\geq \text{DS}/\text{Sa}(T1)\right]=\Phi \left(\frac{\text{ln}\left[Sa(T1,5\%)\right]-\mu }{\sigma }\right)$$

Where,Φstands for standard normal cumulative distribution function, μis the mean value for damage states at various intensity measurements, and σ is the standard deviation for each damage state. As a result, the fragility functions must be developed using two key parameters μ and σ.

## Vulnerability classification and determination of ISVI scores for assessed roads

For several seismic hazard scenarios that are generated from the microzonalization research for the Penang case study, the vulnerability assessment of the roadways and their assets is carried out. According to Sa values, the probability of damage is determined using appropriate fragility functions for Penang roadway systems. Mainly, when evaluating and prioritising the impact of the parameters on the stabilisation of the roadway system, it is important to consider the concept of resistance design for roadway systems. At the collapse stage, the roadway system's destabilisation can be predicted. Two different transmission stages should be examined in order to better understand how this system can be stabilised using the resistance design perception. By examining the % difference/improvement in two different stages based on the damage states and primarily the collapse state at particular collapse intensity measures, it is possible to quantify each parameter's influence on its structural performance and vulnerability behaviour patterns from the fragility functions. The first stage is from the low to moderate state, and the second stage is from the moderate to high state. For the roadway system, interpretation of the Earthquake Resistance Design (ERD) principle can be implemented with respect to the developed Incremental Dynamic Analysis (IDA) and percentage of improvement. The main identified parameters are weighted based on the results, where the most effective parameter is concluded from the probability of damage difference (percentage of improvement) between the two transmission stages at collapse state. The calculation of the ISVI scores is determined using Equation 1, where various ISVI scores are obtained based on the effect of different parameters for the roads. All the parameters with different categories are induced when calculating the ISVI for roadway and its assets.

The vulnerability classification is determined using Equation 1 and is mostly judged based on the weighting of the criteria. The weights are assessed in terms of the percentage improvement obtained from the generated fragility functions. Different earthquake scenarios with varying ground movements are provided and applied to the models to assess the roadway system's susceptibility. The specified ground movements are considered for the most severe conditions with the greatest potential for disaster. Furthermore, the primary goal of this framework is to relate the assessed vulnerability to the accessibility rate, and in order to do so, it is necessary to analyse earthquakes with high seismic intensities ranging from VIII to IX [3]. By considering these important conditions and the validation procedure, the evaluation of roadway vulnerability is expected to be more efficient. Following that, the reduced accessibility values are assessed based on the road vulnerability associated with various earthquake situations. This methodology proposes a new vulnerability categorization based on the predicted ISVI range, which is separated into three primary classes. The road section is divided into three categories: safe (ISVI = 0-0.40), moderately resistant (ISVI = 0.4-0.7), and low resistant (ISVI = 0.7-1) for more information regarding vulnerability classification refer to El-Maissi et al. [5].

**Step 2: traffic disruption assessment approach**

The Accessibility Index (AI) is used to analyse the road network's accessibility disruption. The AI scores show the accessibility rates between different intersecting nodes in the road network, with higher scores reflecting a better accessibility rate. The relative accessibility that investigates the ratio of interconnectivity between different intersecting nodes, should be assessed with respect to distance, travel costs, and travel time in order to determine the AI. The distance is considered the most essential factor in calculating the AI in this method by which, when compared to travel time requirement, it is measured as a constant component with less variations [14,15].

## Locating the key important facilities

**Fig. 7:**
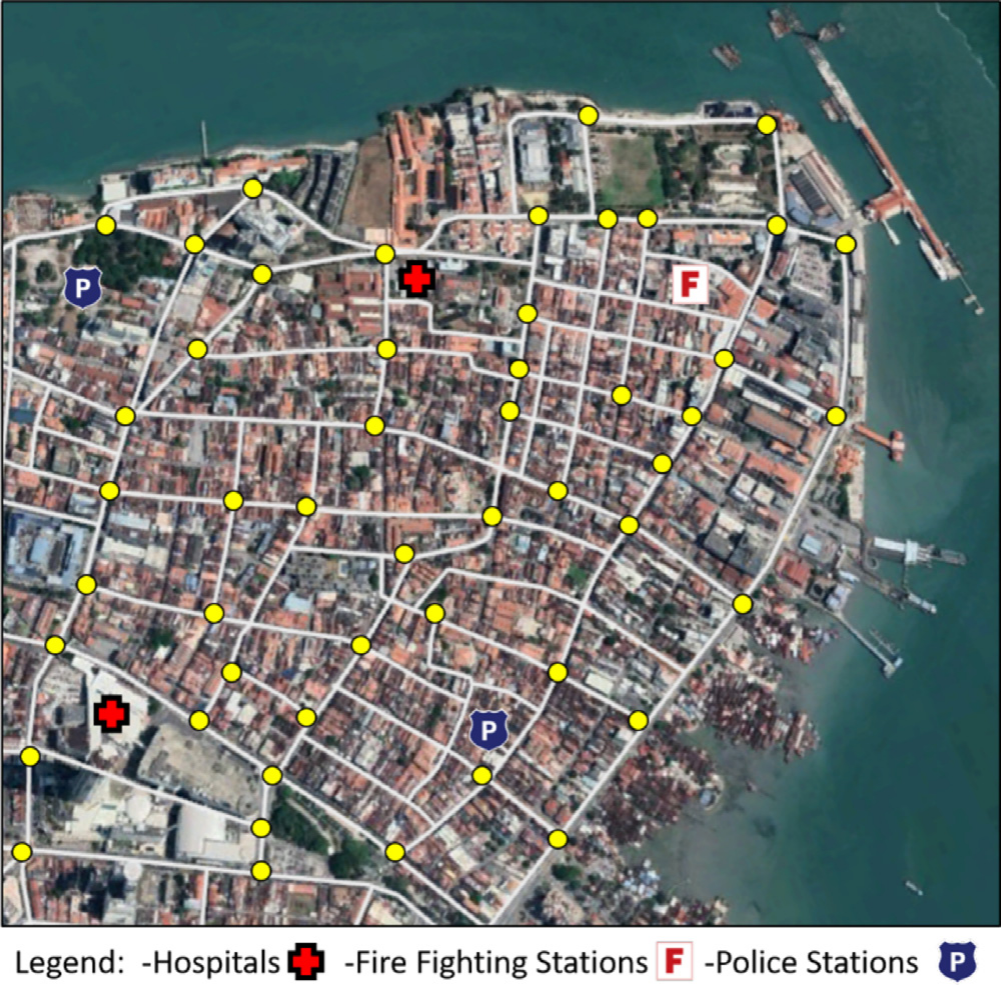
Locating the Important Critical Service Centers in the Investigated Road Network.

The road network and critical service center topologies are created, as well as the critical infrastructure elements and geographical aspects. The critical service centers are classified and located on the map (Hospitals, police stations, and firefighting stations) as shown in Fig. 7. This will help in estimating the relative accessibility and in specifying the emergency and evacuation corridors towards these critical service centers.

## Determination of damaged roads

The goal of this stage is to identify the undamaged corridors, which are the main elements by which, traffic can rely on in the case of an emergency event (emergency corridors). The roads are categorized as safe, moderately resistant, or low resistant depending on the results of the physical damage approach (damaged roads) described in step 1. If there is an available link connecting the intersecting nodes of the road network, the network is characterized as completely linked, and the nodes are regarded equally accessible. On the other hand, when a road network isn't completely connected, it is divided into separate connected sections. The intersecting nodes in these connections may become inaccessible (isolated region) if links are destroyed or obstructed, and the network graph may split up into several elements (road network nodes and links). To locate the isolated regions of the network, a connectivity analysis is performed through the undamaged components (low vulnerable components).

## Accessibility analysis

**Fig. 8:**
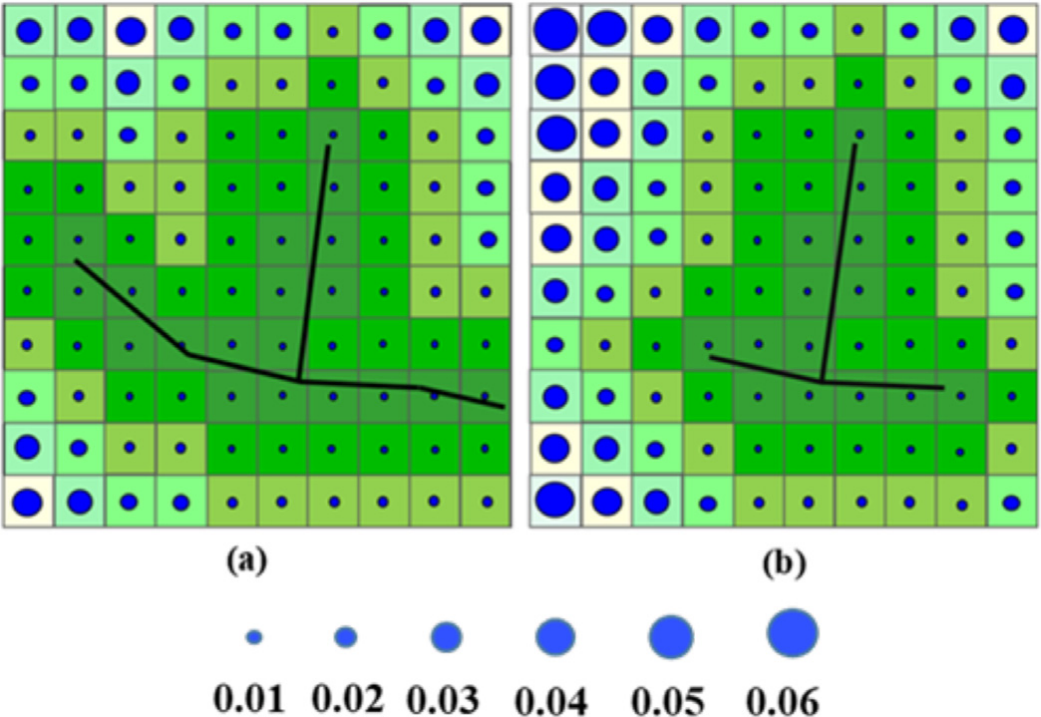
(a) Cost -distance for undamaged network and (b) Cost distance for damaged network. Smaller size of the blue points represents lower cost in terms of travelled distance.

The accessibility assessment is carried out both before and after the earthquake. Distance, cost of travel, and trip time are all terms that may be used to calculate accessibility. The cost-distance analysis is used to generate the accessibility index in this study. The latter is evaluated using ArcGIS to convert a raster grid from a road network vector feature, as seen in Fig.8. The ArcGIS is utilized because it combines decision support technique (AHP) with efficient visualization and mapping, resulting in a strong tool that facilitates land use accessibility mapping [14]. Fig.8 (a) depicts the road before it was destroyed, whereas Fig.8 (b) depicts the road after it is damaged during earthquake. The sources are the cells through which the linkages pass, and the cost distance is calculated by measuring the distance between these sources and other cells, as illustrated in Equations (3) and (4) [15]. The two most important criteria in determining the impedance of the connections are (i) the cost calculated from the distance travelled between origin and destination, and (ii) the course of roadways going through the cells. Therefore, the costs of orthogonal movement between neighboring cells are determined by calculating the average cost of these cells, and the cost of diagonal movement is computed by multiplying the average of the costs between the cells multiplied by √2, to recompense for the longer distance.

Orthogonal cost: $C_{0}=\left(cost1+cost2\right)/2$: (3)
Diagonal cost: $C_{d}=\sqrt{2}\;\left(cost1+cost2\right)/2$: (4)

## Reduced accessibility index

The result of the difference in the two cumulative costs is used to determine reduced accessibility (degree of isolation). Because of the short distance between the source and the cells, the cost distance of the linkages is valued by zero (undamaged). Damaged links, on the other hand, result in higher accessibility ratings, which are scaled depending on accessibility values ranging from 0.00 to 0.06 [15]. Finally, following earthquakes, the accessibility maps for road networks are created using the accessibility index produced by cost-distance analysis on the GIS platform.

**Step 3: Integrated approach**

## Formulate an integrated approach between physical and traffic disruption

The generated damage maps based on Intrinsic Seismic Vulnerability Index (ISVI) and the Accessibility Index (AI) approach have shown that it is important to reflect the correlation between the physical damage and traffic disruption, because the generated integrated damage maps are showing the main accessible roads. This is determined by the evaluated physical damage that is affecting roads during earthquakes, aiming at identifying the critical service centers that are expected to have higher accessibility, and hence, should be used considered in the emergency planning. This correlation can be achieved by developing the built emergency accessibility GIS maps that are described in the following section.

## Building emergency accessibility GIS maps

This process includes the creation and visualization of a transportation performance map using the ArcGIS platform, with the objective of determining the primary corridors that will be operative following earthquakes. As explained in step 2, the retrieved data is displayed as ArcGIS maps to show the accessibility for the investigated areas. The integrated maps show (1) damaged roadways, (2) accessibility for each district in the designated region, and (3) the primary emergency pathways that will be operational following earthquakes.

Finally, based on the created integrated approach between traffic functionality and physical damage assessment for the road network for all the investigated roadways, a link between the ISVI and AI is drawn. The visualized results will help stakeholders and decision-makers to figure out which parts of the city are expected to have lower accessibility scores to emergency service centers during earthquakes, allowing for additional preventative steps and re-maintenance operations in the near future. These steps are anticipated to make the network more accessible, resulting in more resilient cities.

## Method validation

A preliminary study is developed in Penang area, Malaysia to validate the present methodology, based on the research by Adafer and Bensaibi [3] that tackled the Intrinsic Seismic Vulnerability Index (ISVI) for roadways and their assets with respect to different empirical methods. For measuring the ISVI, the researchers used a variety of vulnerability parameters and weighting variables. In addition, both researchers employed the judgmental expert technique to weight the primary characteristics included in the ISVI. The current framework builds-on previous models by using an analytical assessment based on NLDA to provide a novel weighting technique for the vulnerability metrics for road structural elements.

**Fig. 9:**
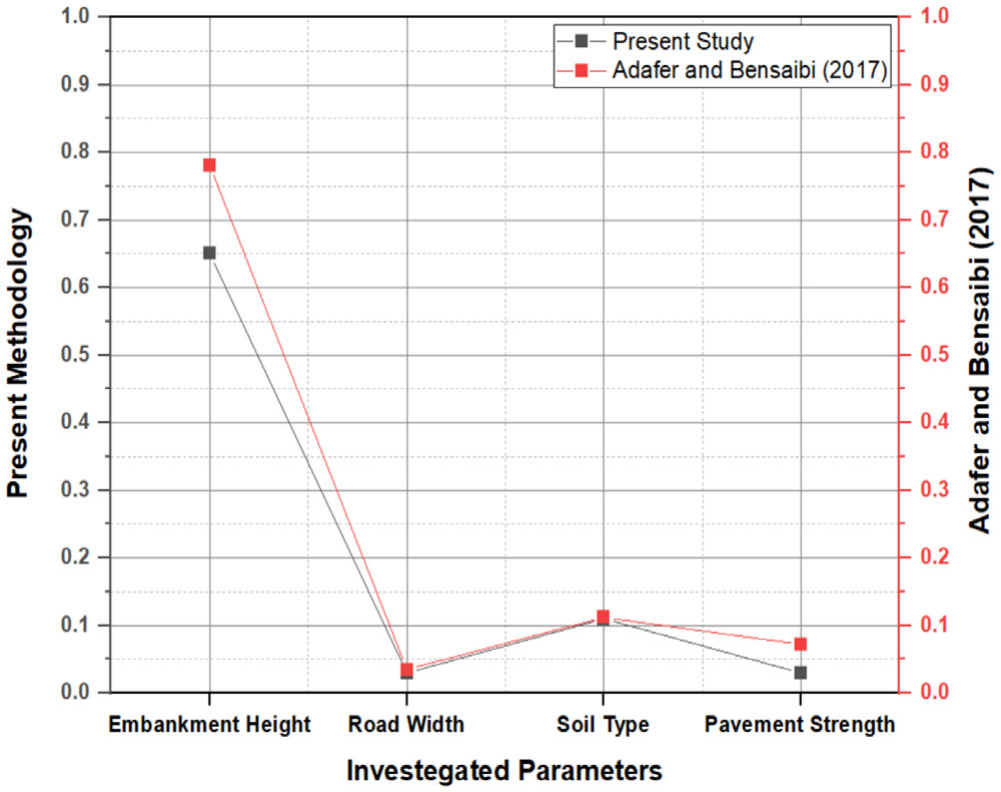
Variation between the Weighting Scores of the Investigated Parameters when Comparing the Present Methodology with Adafer and Bensaibi.

For the Penang case study, a vulnerability assessment of the roadway and its assets is carried out for several seismic hazard scenarios established from the micro-localization research [13,16]. The height of the embankment, pavement strength, road width, and soil type are the four major factors used to classify roadway systems. The likelihood of damage is assessed using Non-Linear Dynamic Analysis (NLDA) for the roadway and its assets usually found in Penang in terms of spectrum acceleration, as defined in the Malaysian National Annex. The weighting values vary because this study uses an analytical methodology to conduct the ISVI and weight the primary characteristics, whereas previous studies relied mostly on expert judgement approaches. Although there is a little difference between the researched weighting scores of the investigated parameters when comparing the results of this study to those of Adafer and Bensaibi [3] the priority of the weighted characteristics based on their impact on the roadway systems and their assets remains compatible as illustrated in Fig.9.

The most significant parameter, embankment height, appears to have by far the highest percentage of improvement, followed by the number of lanes, while the soil type and pavement strength are the least influential criteria, with the soil type having a tiny benefit. ISVI is primarily concerned with the individual qualities of roadway infrastructures, but it is unaffected by external influences. The number of elements utilized depends on the type of vulnerability being investigated and the major context of this ISVI, where weighting factors are typically used to analyse the relevance of different aspects to road vulnerability. Due to the obvious wide range of outcomes, it is critical to provide this methodology that is considered a more accurate ISVI assessment method.

## Conclusions

The proposed methodology works on assessing the seismic vulnerability of roadways and their assets. A modified Intrinsic Seismic Vulnerability Index (ISVI) approach is conducted based on previous studies that tackled this issue, where the ISVI scores are considered the baseline of the vulnerability assessment of roadway systems and to extract the accessibility rates. These ISVI scores are integrated with the Accessibility Index (AI) approach that is conducted on the basis of geographical aspects. The integration between the physical and traffic approach is making this methodology more significant, since most of the past studies are focusing on single criterion (Functionality or physical damage or network traffic) without considering the integration between these different assessment factors and approaches. In real-world scenarios, the integrated model may aid in the discovery of correlations between all assets of road networks, as well as the development of a disaster management tool to prevent fatalities and economic losses during disasters. Future research should focus on developing well-informed disaster management maps, which include the most effective placements for vital service centers and focus on the interdependencies between various infrastructures, such as electricity, water, transit, airports, and fuel, as well as the pace of interaction between transportation networks and other systems. Finally, the proposed framework focus on road embankments and pavements; however, its application can be extended to other assets such as bridges, tunnels, and retaining walls in future studies.

## Declaration of Competing Interest

The authors declare that they have no known competing financial interests or personal relationships that could have appeared to influence the work reported in this paper.

## Data Availability

No data was used for the research described in the article.

## References

[1] Argyroudis S.A., Mitoulis S.Α., Winter M.G., Kaynia A.M. (2019). Fragility of transport assets exposed to multiple hazards: State-of-the-art review toward infrastructural resilience. Reliab. Eng. Syst. Saf..

[2] Kilanitis I., Sextos A. (2019). Integrated seismic risk and resilience assessment of roadway networks in earthquake prone areas. Bull. Earthquake Eng..

[3] Adafer S., Bensaibi M. (2017). Seismic vulnerability classification of roads. Energy Procedia.

[4] Zakaria N.M., Yusoff N.I.M., Hardwiyono S., Mohd Nayan K.A., El-Shafie A. (2014). Measurements of the stiffness and thickness of the pavement asphalt layer using the enhanced resonance search method. Sci. World J..

[5] El-Maissi A.M., Argyroudis S.A., Nazri F.M. (2020). Seismic vulnerability assessment methodologies for roadway assets and networks: a state-of-the-art review. Sustainability.

[6] Maruyama Y., Yamazaki F., Mizuno K., Tsuchiya Y., Yogai H. (2010). Fragility curves for expressway embankments based on damage datasets after recent earthquakes in Japan. Soil Dyn. Earthquake Eng..

[7] Silva V. (2019). Current challenges and future trends in analytical fragility and vulnerability modeling. Earthquake Spectra.

[8] Vamvatsikos D., Cornell C.A. (2005). Developing efficient scalar and vector intensity measures for IDA capacity estimation by incorporating elastic spectral shape information. Earthquake engineering & structural dynamics.

[9] Kassem M.M., Nazri F.M., Farsangi E.N., Tan C.G. (2021).

[10] Cordova P.P., Deierlein G.G., Mehanny S.S., Cornell C.A. (2000). The Second US-Japan Workshop on Performance-Based Earthquake Engineering Methodology for Reinforced Concrete Building Structures.

[11] Shome N., Cornell C.A., Bazzurro P., Carballo J.E. (1998). Earthquakes, records, and nonlinear responses. Earthquake spectra.

[12] Ibrahim Y.E., El-Shami M.M. (2011). Seismic fragility curves for mid-rise reinforced concrete frames in Kingdom of Saudi Arabia. IES J. Part A: Civil Struct. Eng..

[13] Kassem M.M., Nazri F.M., Farsangi E.N. (2020). The seismic vulnerability assessment methodologies: a state-of-the-art review. Ain Shams Eng. J..

[14] Marinoni O. (2004). Implementation of the analytical hierarchy process with VBA in ArcGIS. Comput. Geosci..

[15] Bono F., Gutierrez E. (2011). A network-based analysis of the impact of structural damage on urban accessibility following a disaster: the case of the seismically damaged Port Au Prince and Carrefour urban road networks. J. Transp. Geogr..

[16] bin Rambat S., Shi Z., bin Mazlan S.A. (2021). Seismic vulnerability assessment in Ranau, Sabah, using two different models. ISPRS Int. J. Geo-Inf..

